# Localization of HPV-18 E2 at Mitochondrial Membranes Induces ROS Release and Modulates Host Cell Metabolism

**DOI:** 10.1371/journal.pone.0075625

**Published:** 2013-09-24

**Authors:** Deborah Lai, Chye Ling Tan, Jayantha Gunaratne, Ling Shih Quek, Wenlong Nei, Françoise Thierry, Sophie Bellanger

**Affiliations:** 1 Papillomavirus Regulation and Cancer, Institute of Medical Biology, Agency for Science, Technology and Research (A*STAR), Singapore, Singapore; 2 Quantitative Proteomics Group, Institute of Molecular and Cell Biology, Agency for Science, Technology and Research (A*STAR), Singapore, Singapore; Louisiana State University Health Sciences Center, United States of America

## Abstract

Papillomavirus E2 proteins are predominantly retained in the nuclei of infected cells, but oncogenic (high-risk) HPV-18 and 16 E2 can shuttle between the host nucleus and cytoplasm. We show here that cytoplasmic HPV-18 E2 localizes to mitochondrial membranes, and independent mass spectrometry analyses of the E2 interactome revealed association to the inner mitochondrial membrane including components of the respiratory chain. Mitochondrial E2 association modifies the cristae morphology when analyzed by electron microscopy and increases production of mitochondrial ROS. This ROS release does not induce apoptosis, but instead correlates with stabilization of HIF-1α and increased glycolysis. These mitochondrial functions are not shared by the non-oncogenic (low-risk) HPV-6 E2 protein, suggesting that modification of cellular metabolism by high-risk HPV E2 proteins could play a role in carcinogenesis by inducing the Warburg effect.

## Introduction

Infection of the ano-genital tract epithelium by Human Papillomaviruses (HPV) leads to development of benign and malignant lesions, with HPV DNA detected in close to 100% of cervical cancers [1]. Expression of E6 and E7, the two HPV viral oncogenes, is negatively regulated by E2 in benign lesions [2,3]. During the carcinogenesis process, the E2 ORF is usually disrupted by viral DNA integration into the host cell DNA, allowing E6/E7 expression and transformation. E2 proteins from oncogenic HPV only (called high-risk, by opposition to low-risk HPV which can only induce benign lesions) have been shown to actively shuttle between the nucleus and the cytoplasm, where E2 accumulation mediates apoptosis [4]. However, beyond these 2 obvious anti-proliferative functions, high-risk HPV E2 proteins have the property to induce chromosomal instability and DNA breaks in mitosis [5]. This phenomenon, specific to high-risk HPV E2 proteins compared to low risk ones, has been proposed to facilitate integration of the HPV genome into the host cell genome. Moreover, the high-risk HPV-18 E2 protein stabilizes Skp2 through a mechanism involving E2-mediated inhibition of APC/C, thus pushing the cells faster towards the G1/S transition [6], similarly to E7. More recently, E2 from HPV-8 (skin oncogenic HPV) has been shown to be able to induce tumors in mice [7]. Therefore, although historically E2 was rather classified amongst viral “anti-oncogenes”, these recent data unambiguously indicate that E2 proteins from high-risk HPV do have some oncogenic characteristics [8].

Metabolism is deeply modified in cancer cells, one frequent phenomenon being a shift from respiration (mediated through mitochondria) to aerobic glycolysis (occurring in the cytoplasm), also known as “Warburg effect” [9]. Aside from their role in inducing apoptosis, mitochondria are involved in the aerobic respiration process, also called oxidative phosphorylation or OXPHOS. The mitochondrial inner membrane houses the electron transport chain, which comprises 5 distinct complexes, and produces the majority of cellular ATP under aerobic conditions. The first two electron transport complexes, NADH dehydrogenase (complex I) and fumarate reductase (complex II), oxidize NADH and FADH2 respectively, and transfer the resultant electrons to cytochrome bc1 (complex III) via the ubiquinol intermediary. Cytochrome c then transports electrons from complex III to cytochrome c oxidase (complex IV), which subsequently uses them to reduce oxygen to water. Each electron shift in this sequence produces energy which transfers protons into the intermembrane space, creating an electrochemical gradient eventually used by the ATP synthase (complex V) to produce ATP. However, mitochondrial respiration is also a major source of intracellular reactive oxygen species (ROS) which can cause oxidative cell damage. Indeed, a small proportion of electrons leaks from OXPHOS complexes (primarily I and III) and interacts with molecular oxygen to generate O_2_^−^· (superoxide anion), which is the predominant ROS in mitochondria and acts as a precursor for most other ROS. Under normal conditions, anti-oxidant cellular defenses are sufficient to maintain ROS concentrations at non-toxic levels despite the ongoing production of O_2_^−^· by mitochondria. However, increased leakage of electrons from respiratory complexes, due to dysfunction of one of them or down-regulation of ROS scavengers and anti-oxidant enzymes, can overcome these defense mechanisms and induce cellular stress.

In the current report, we use immunofluorescence and cellular fractionation approaches to demonstrate that the cytoplasmic fraction of the high-risk HPV-18 E2 protein localizes to mitochondrial membranes. Mass spectrometry analyses, obtained independently, indicate that ~12% of proteins that interact with HPV-18 E2 are of mitochondrial origin, and include key mediators of the OXPHOS process. In contrast, the low-risk HPV 6 E2 protein, which exhibits a more nuclear localization, shows milder mitochondrial interactions. Expression of HPV-18 E2, but not HPV-6 E2, modifies the cristae morphology and augments mitochondrial release of ROS without inducing cell death. Increased ROS correlates with enhanced expression of the transcription factor HIF-1α and activation of select HIF-1 target genes. Consecutively, we detected an increase in glycolysis characterized by lactate production in E2-expressing cells. Altogether, these data strongly suggest induction of the Warburg effect by high-risk HPV E2 proteins.

## Results

### HPV-18 E2 localizes at mitochondria

**Figure 1:**
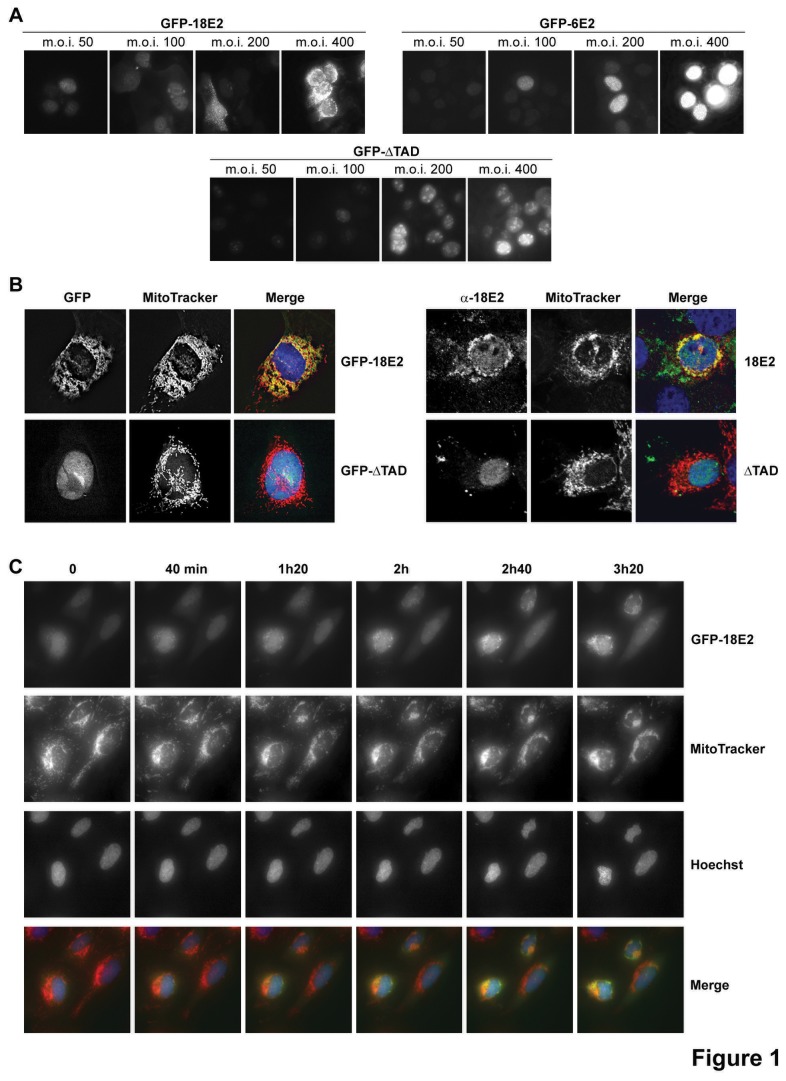
Cytoplasmic HPV-18 E2 protein localizes at mitochondria. (A) Localization of GFP-18E2, GFP-6E2 and GFP-ΔTAD in HaCaT cells infected with increasing m.o.i. of the corresponding recombinant adenoviruses (Ad). (B) Left panel: AdGFP-18E2 and AdGFP-ΔTAD-infected cells (m.o.i. 200) labeled with MitoTracker red. Right panel: Cells transfected with untagged HPV-18 E2 and ΔTAD expression vectors, labeled with anti-HPV-18 E2 antibody and MitoTracker red. (C) Time-lapse microscopy analyses of Saos-2 living cells (osteosarcoma cells used here for their large cytoplasm) infected with AdGFP-18E2 (m.o.i. 200), labeled with MitoTracker red and Hoechst 33342.

High-risk HPV-16 and 18 E2 proteins have previously been shown to be partially localized in the cytoplasm whereas low-risk HPV-6 and 11 E2 proteins appeared more nuclear [4]. Since keratinocytes are host cells for HPV, we infected HaCaT cells (HPV-negative immortalized human keratinocytes) with increasing concentrations of a recombinant adenovirus that expressed the HPV-18 GFP-E2 protein (GFP-18E2), to show that HPV-18 E2 gradually leaves host cell nuclei as expression levels increase, to eventually localize in cytoplasmic speckles at m.o.i. (multiplicity of infection) 200 and 400. The low risk HPV-6 E2 protein needs a much higher level of nuclear expression before appearing partially localized to the cytoplasm (cytoplasm localization visible from m.o.i. 400). A control protein that lacks the transactivation domain of 18E2 (GFP-ΔTAD) exhibits a localization similar to 6E2 (Figure 1A).

MitoTracker Red labeling revealed that after infection at m.o.i. 200, these HPV-18 E2 cytoplasmic speckles co-localized with mitochondria (Figure 1B, left panel), but not with other cytoplasmic organelles (Figure S1). We verified by immunofluorescence, using an antibody against HPV-18 E2, that the untagged wild-type HPV-18 E2 also localized at mitochondria (Figure 1B, right panel). Time-lapse microscopy established that mitochondria did not migrate towards E2, but that E2 gradually localized to mitochondria. As shown in Figure 1C, E2 initially accumulates in the nucleus up to a threshold level, then shuttles to the cytoplasm and immediately re-localizes to mitochondria.

### Mass spectrometry analyses revealed association of high-risk E2 with both inner and outer mitochondrial membranes

**Figure 2:**
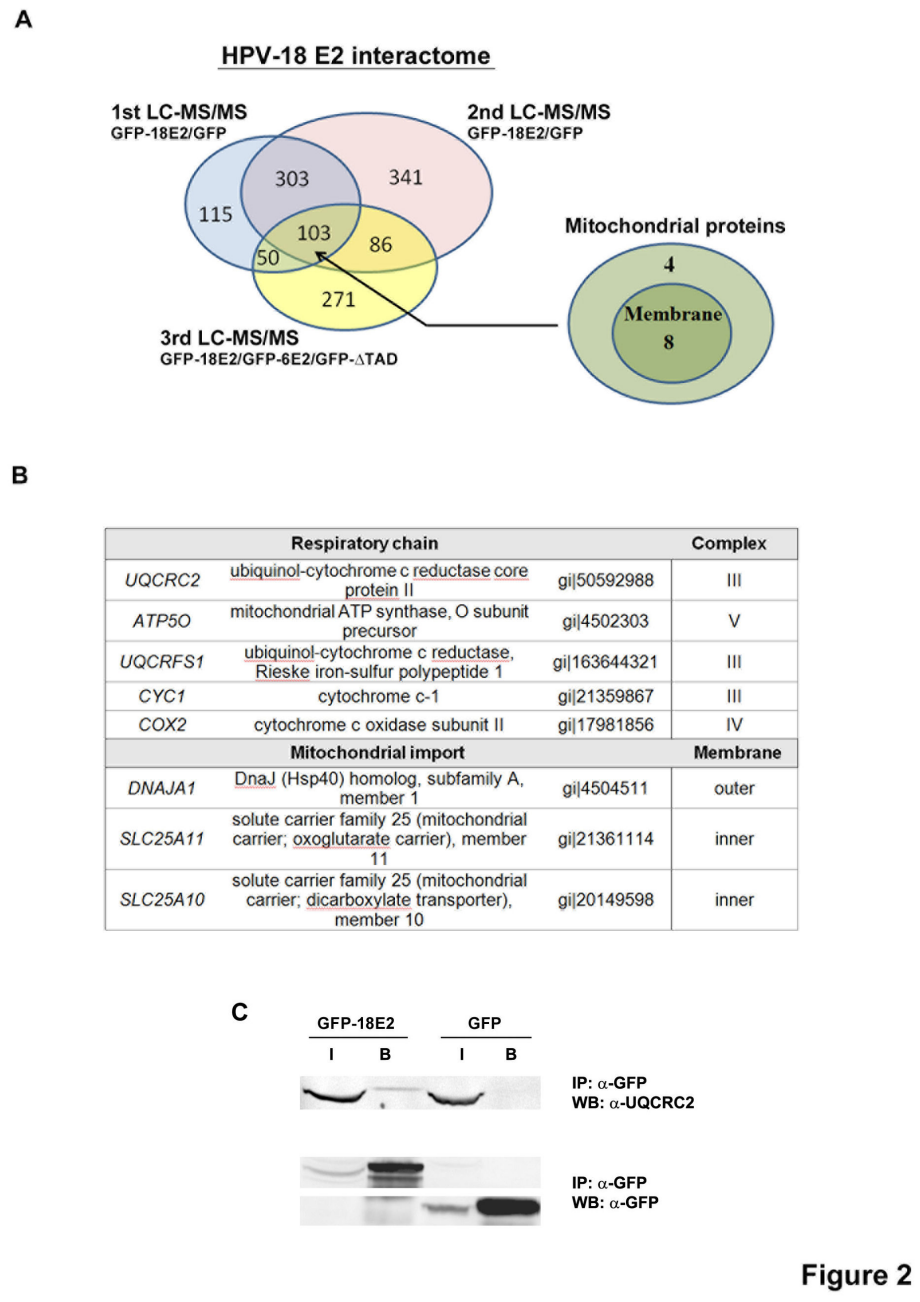
HPV-18 E2 interactome reveals mitochondrial membrane proteins. (A) Venn diagram established from 3 LC-MS/MS independent experiments, including 2 replicates. (B) List of mitochondrial membrane proteins interacting with HPV-18 E2 found in the 3 independent MS experiments. (C) Co-immunoprecipitation confirming interaction of HPV-18 E2 with UQCRC2. I = input (1/50), B = beads.

An independent way of investigating and confirming E2 localization at mitochondria was to immunoprecipitate (IP) HPV-18 E2 complexes and to subject them to mass spectrometry (MS) analyses. The extract from cells expressing Flag-GFP-18E2 (m.o.i. 20) was used as bait containing sample, whereas extract from cells expressing Flag-GFP alone (m.o.i. 20) was used as a control. Tandem Affinity Purification (TAP, double IP) against Flag (1^st^ IP) and GFP (2^nd^ IP) was carried out with 2 biological replicates (independent experiments) to capture E2 associated proteins. The proteins detected in the bait sample (Flag-GFP-E2) with 2 or more unique peptides and at least 2 spectral counts for both replicates, while no peptide was detected in the GFP control sample (GFP IP spectral counting must be 0), were considered as proteins specifically associated to E2. The analysis of the intersection between the 2 replicates gave us around 400 potential E2 interactants. A similar experiment using Flag-GFP-ΔTAD as a control, instead of Flag-GFP, was used to further prioritize this list. For this experiment, a single IP was carried out using Flag-GFP-18E2 (bait) and Flag-GFP-ΔTAD (control) extracts. Proteins detected in the Flag-GFP-E2 bait sample with at least 2 unique peptides and with a ratio (spectral counts of Flag-GFP-E2 IP/spectral counts of Flag-GFP-ΔTAD IP) ≥ 2 were considered as associated proteins to the TAD domain of E2. The Venn diagram (Figure 2A) shows the numbers of E2-associated proteins in each experiment and intersections between experiments. The intersection between the 3 MS experiments revealed 103 proteins (Figure 2A). Amongst these proteins, 12 (~12%) were mitochondria-associated proteins, most of them being proteins associated with mitochondrial membranes (Figure 2A). Figure 2B shows the list of mitochondrial membrane proteins found to interact with HPV-18 E2 in the 3 MS (see Table S1 for the list of all mitochondrial proteins found to interact with HPV-18 E2). The main E2 interactants found were proteins from the inner membrane, more than half being involved in the OXPHOS process (mainly complex III). We attempted to detect some of the mitochondrial membrane interactants identified by MS using immunoprecipitation followed by western-blot, instead of immunoprecipitation followed by MS. This approach was successful with the UQCRC2 protein (Figure 2C), one of the strongest HPV-18 E2 interactants, but not with the other mitochondrial membrane proteins tested. This is probably due to the insoluble characteristics of most mitochondrial membrane proteins and to the reduced sensitivity of western-blot compared to MS.

Although the relative importance of E2 interactions with each of these mitochondrial proteins remains to be determined, these results clearly suggested that E2 could be found associated with mitochondrial membranes. Importantly, all MS experiments were performed after adenoviral infection at low m.o.i. (20) to avoid excess and mimic levels of HPV-18 E2 expressed from HPV episomes, which were found to be similar to those obtained after infection at m.o.i. 50 (Figure S2). In these conditions, HPV-18 E2 appears exclusively nuclear by microscopy (see Figure 1, m.o.i. 50, for comparison), and yet, MS allowed us to identify mitochondrial partners. This indicates that localization of HPV-18 E2 to mitochondria occurs prior to visualization by microscopy, but can be detected by ultra sensitive methods like MS. Therefore, we reasoned that despite the fact that the low-risk HPV-6 E2 protein was not visibly cytoplasmic and/or associated to mitochondrial speckles in microscopy at m.o.i. 20, it could still be partly localized at mitochondria. An IP of Flag-GFP-6E2 was included in the 3^rd^ LC-MS/MS experiment, together with Flag-GFP-18E2 and Flag-GFP-ΔTAD (as a control), allowing us to directly compare HPV-18 and HPV-6 E2 interactomes. As shown in blue in Table S1, only 2 out of the 12 HPV-18 E2 mitochondrial partners were found to interact with HPV-6 E2 and with less efficiency. Ten partners were specific to HPV-18 E2 (in red).

### Cell fractionation confirmed the mitochondrial membrane localization of HPV-18 E2

**Figure 3:**
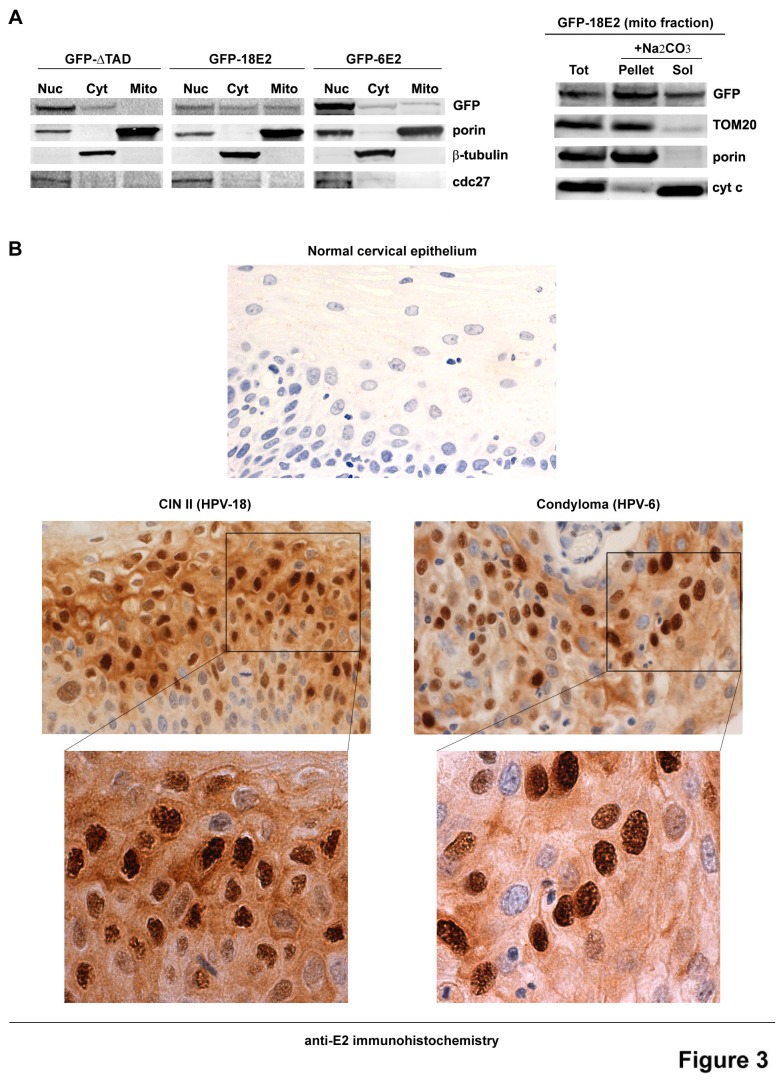
HPV-18 E2 localizes to mitochondrial membranes. (A) Left panel: Western-blot analyses after fractionation of extracts from HaCaT cells infected with AdGFP-ΔTAD, AdGFP-18E2, and AdGFP-6E2. Nuc: nuclear fraction, Cyt: cytoplasmic fraction, Mito: crude mitochondrial fraction after Percoll gradient. Porin, β-tubulin and cdc27 were used as markers of mitochondria, cytoplasm and nucleus respectively. Right panel: The crude mitochondrial fraction was submitted to alkali extraction. Tot: total crude mitochondria before alkali extraction. Pellet: pellet after alkali extraction containing membrane-bound proteins, Sol: supernatant after alkali extraction containing soluble proteins. (B) Immunohistochemistry showing localization of HPV-18 E2 in CIN II (left panels) and HPV-6 E2 in condyloma (right panels) using the anti-HPV-16 E2 antibody. The lower panels are high magnifications of upper panels. The first upper panel shows labeling of a HPV-negative normal cervical epithelium with the same anti-HPV-16E2 antibody performed in the same conditions as the HPV-positive samples.

To better define HPV-18 E2 mitochondrial distribution and confirm its membrane localization, we fractionated extracts of HaCaT cells expressing GFP-ΔTAD, GFP-18E2 or GFP-6E2 (Figure 3A, left panel). For this experiment, an m.o.i. of 200 was used to be able to detect sufficient quantity of E2 by western-blot in the different fractions. GFP-18E2 was found in both nucleus and cytoplasm, while GFP-6E2 and GFP-ΔTAD were predominantly nuclear at similar levels of expression. As expected from immunofluorescence and MS, GFP-18E2 was detected in the mitochondrial fraction together with the porin membrane transport protein, while GFP-ΔTAD was absent from that fraction and only a very weak quantity of GFP-6E2 could be found associated with mitochondria (Figure 3A, left panel). After alkali extraction of pure mitochondria using Na _2_CO_3_ (Figure 3A, right panel), GFP-18E2 could be detected in both the pellet (containing membrane-bound proteins like TOM20 [outer membrane] and porin [inner membrane]), and the supernatant soluble fraction to a lesser extent (this fraction contains soluble proteins and proteins weakly associated with membranes, like cytochrome c). Together with the immunofluorescence and MS experiments, this new data led us to conclude that at least part of HPV-18 E2 was associated with mitochondrial membranes.

### HPV-18 E2 is both cytoplasmic and nuclear in vivo in CIN

In order to determine whether the cytoplasmic localization of HPV-18 E2 also occurs in vivo, a CIN II lesion associated with HPV-18 was stained by immunohistochemistry with an anti-16E2 antibody (homemade and raised against the C-terminal part of HPV-16 E2) cross-reacting with E2 proteins from different genotypes, including HPV-18 and 6. A large proportion of HPV-18 E2 could be found in the cytoplasm of CIN II cells (Figure 3B left panel), whereas in the same conditions and at similar levels of E2 staining intensity, a HPV-6-positive condyloma showed a much stronger nuclear localization of HPV-6 E2 (Figure 3B, right panel), thus validating the in vitro data. A normal cervical epithelium was used as a negative control where the anti-16E2 antibody gave no signal (Figure 3B, upper panel).

### HPV-18 E2 induces loss of cristae structure

**Figure 4:**
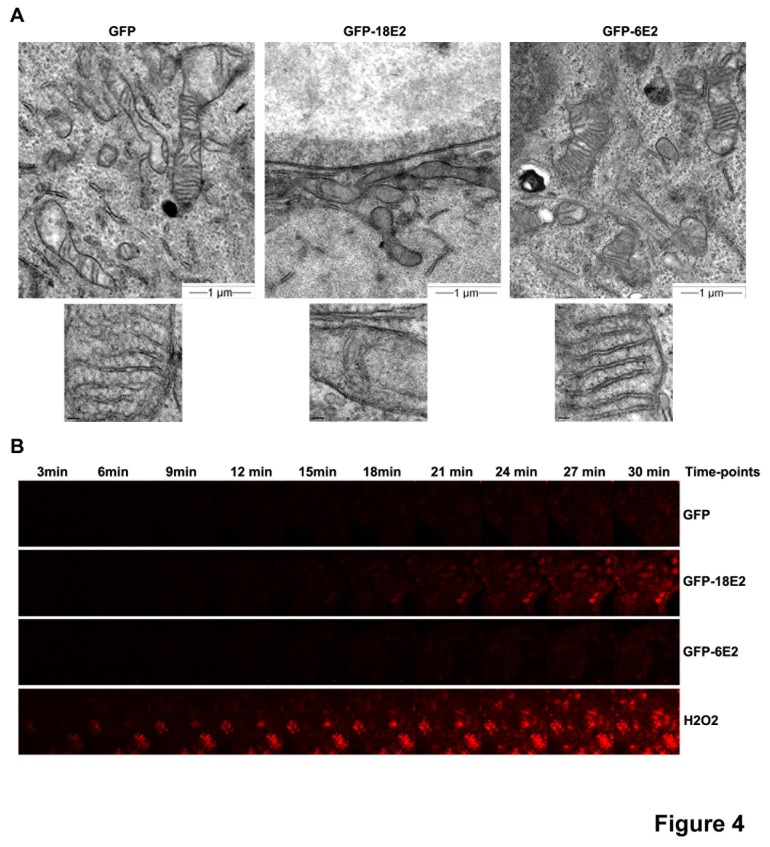
HPV-18 E2 modifies cristae structure and increases release of mitochondrial ROS. (A) Electron microscopy showing the structure of mitochondria of HaCaT cells expressing GFP, GFP-18E2 or GFP-6E2. Higher magnification pictures show the internal structure of the mitochondrial cristae. (B) Time-lapse experiments showing mitochondrial ROS production (MitoSOX labeling) in cells infected with AdGFP-E2 (HPV-18 and HPV-6) or AdGFP during 30min after beginning of MitoSOX treatment. H_2_O_2_ treated cells were used as ROS positive controls.

The inner membrane of mitochondria contains folds forming a structure called cristae which enhances the inner membrane surface. Since HPV-18 E2 interacts with various components of the mitochondrial inner membrane, including ATP synthase involved in cristae morphology [10], we decided to look at the structure of mitochondria by electron microscopy and more specifically we analyzed the cristae in GFP-18E2- and GFP-6E2-expressing cells compared to GFP-expressing control cells (m.o.i. 200). As shown in Figure 4A, mitochondria in GFP-18E2-, GFP-6E2- and GFP-expressing cells did not exhibit obvious differences in size or global number per cell. However, in GFP-18E2-expressing cells, mitochondria tended to aggregate closer to the nuclei, and displayed a reduced cristae compartment, some mitochondria being totally devoid of cristae. Nevertheless, we could still discern a double mitochondrial membrane, indicating that the overall structure of mitochondria was conserved in the presence of HPV-18 E2 (Figure 4A, higher magnification, middle panel). In GFP-6E2-expressing cells, although some mitochondria exhibited less cristae, most of them looked similar to the GFP control (Figure 4A, compare left and right panels).

### HPV-18 E2 induces mitochondrial ROS release

Mitochondrial morphology is crucially linked to energy metabolism. The way the cristae morphology directly affects the efficiency of OXPHOS and/or vice-versa is not clear, but enhanced respiration correlates with enlarged cristae compartment, whereas low OXPHOS and high glycolysis are associated with smaller mitochondria with reduced cristae number [11]. We thus decided to examine whether E2 affected OXPHOS by analyzing mitochondrial generation of ROS, which are released in case of dysfunction of the OXPHOS process [12]. Using MitoSOX Red, which labels superoxide anion produced by mitochondria, we detected by direct fluorescence (30 minutes time-lapse experiments) that GFP-18E2-expressing HaCaT cells (m.o.i. 20), but not GFP-6E2-expressing cells, produced higher levels of superoxide than GFP-expressing cells (Figure 4B). Analysis of red fluorescence on slides (Figure S3A) and flow-cytometry (Figure S3B) were used to verify and quantify the production of superoxide, which was found about 2 to 3-fold higher (depending on experiments) in GFP-18E2-expressing cells than in GFP-expressing cells, while it was comparable to positive control cells treated with H_2_O_2_ (Figure S3B). Since this method only detects mitochondrial superoxide (excluding other cellular ROS), which is known to be generated as a by-product of OXPHOS, increase in MitoSOX Red labeling following HPV-18 E2 expression infers that HPV-18 E2 induces dysfunction of the OXPHOS process.

### HPV-18 E2 localization at mitochondria does not induce apoptosis

**Figure 5:**
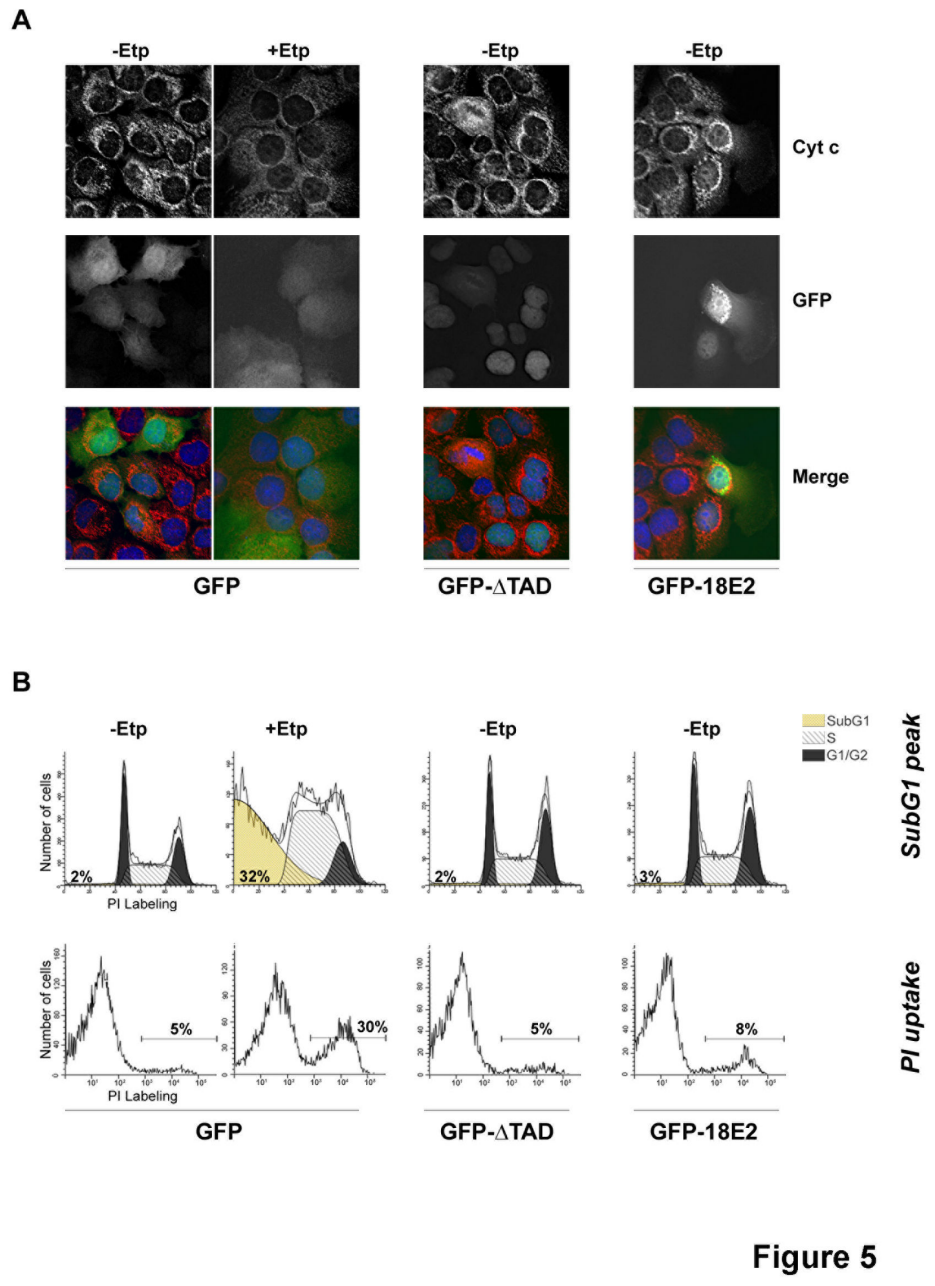
Localization of HPV-18 E2 at the mitochondria does not induce apoptosis. (A) Cytochrome c labeling (cyt c) in HaCaT cells infected with AdGFP, AdGFP-ΔTAD or AdGFP-18E2. +Etp = +Etoposide, -Etp = +DMSO. Merge pictures show cytochrome c in red, GFP in green and DNA (DAPI) in blue. (B) Flow-cytometry analyses of the same samples showing the sub-G1 and PI uptake-positive populations characteristic of cell death. The data shown are representative of more than 5 independent experiments.

Since ROS are potential inducers of apoptosis, and inversely apoptosis can generate superoxide [13], it was necessary to test out whether apoptosis was activated in our conditions. Although high-risk E2 proteins have been shown to induce the extrinsic pathway of apoptosis through binding to caspase 8 when highly expressed only [14], it remained possible that translocation of HPV-18 E2 to mitochondria could modulate the intrinsic pathway of apoptosis in our conditions of low expression of E2. Since cytochrome c release from mitochondria is a marker of this pathway, we assayed the release of cytochrome c in cells expressing GFP-18E2, GFP-ΔTAD or GFP (m.o.i. 100), 28h after infection. Etoposide treatment in GFP-expressing cells was used as a positive control for apoptosis and generated massive release of cytochrome c, characterized by a diffuse signal in the cytoplasm (Figure 5A). In contrast, GFP-18E2-expressing cells did not show evidence of cytochrome c release, even the rare ones expressing high levels of E2 visibly associated with mitochondria (Figure 5A). These data are further supported by the fractionation experiments described above which suggested that cytochrome c was not released in the cytoplasm of GFP-18E2-expressing cells, even when infected at higher m.o.i. (Figure S4 for western-blot against cytochrome c, m.o.i. 200, related to Figure 3A).

In addition, flow-cytometry analyses of the same samples failed to reveal a sub-G1 population characteristic of apoptosis (either induced by the extrinsic or intrinsic pathway), or a significant population positive for propidium iodide uptake (which labels both apoptotic and necrotic cells) in GFP-18E2-expressing cells (Figure 5B). Conversely, etoposide treatment of GFP-expressing cells induced a large sub-G1 peak (32%) as well as marked PI uptake (30%). These experiments show that low levels of HPV-18 E2 (up to m.o.i. 200) do not induce cells death in our system despite E2 interaction with mitochondria.

### HPV-18 E2 increases HIF-1α protein levels and glycolysis

**Figure 6:**
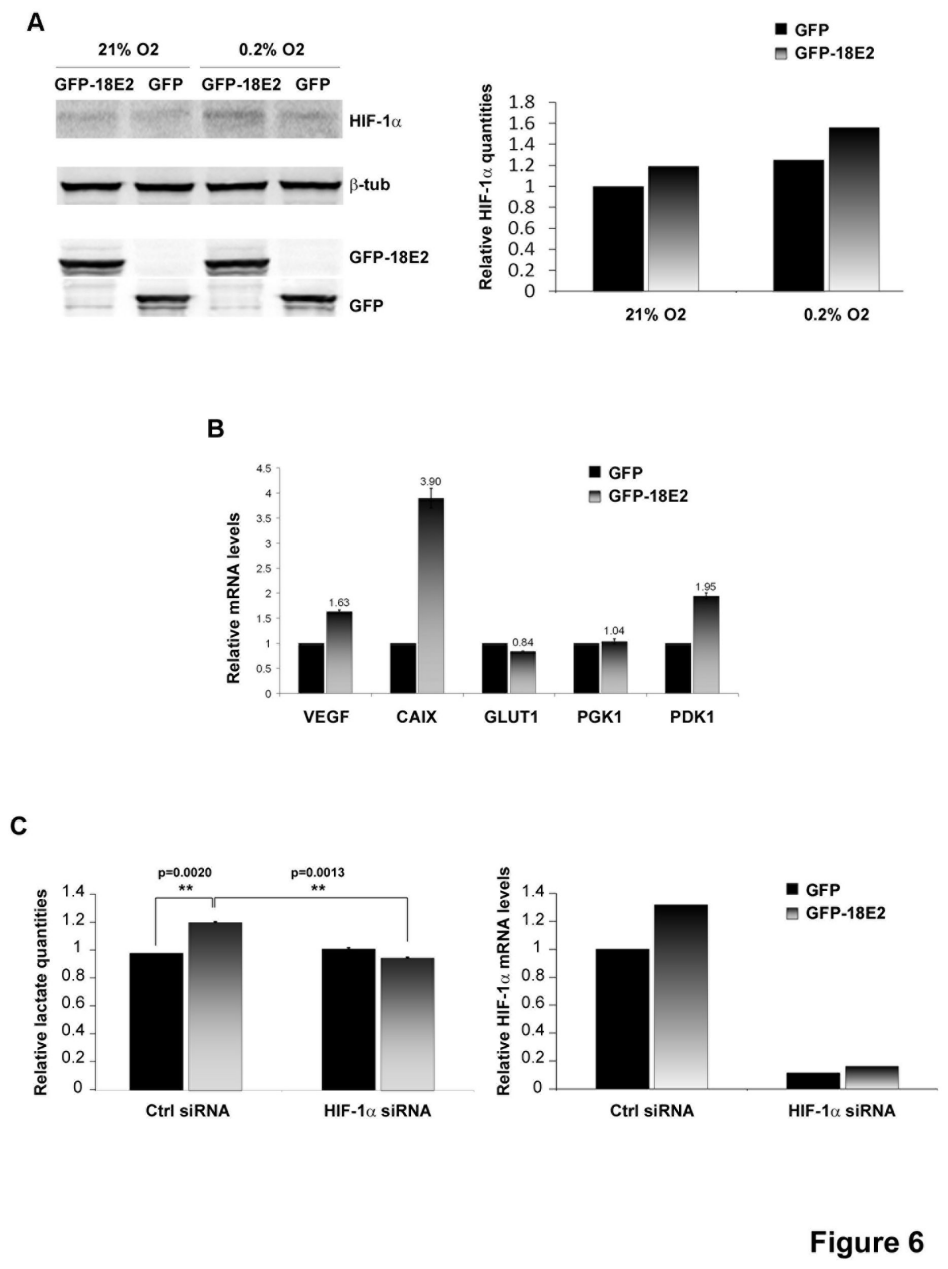
HPV-18 E2 modulates host cell metabolism. (A) Left panel: Western-blot analyses showing stabilization of HIF-1α in HaCaT cells infected with AdGFP-18E2 compared to AdGFP-infected cells and cultured in 21% O_2_ (normoxia) or 0.2% O_2_ (hypoxia). Right panel: Quantification of the western-blot using ImageQuant. (B) Real-time PCR from cDNAs of HaCaT cells infected with AdGFP-18E2 or AdGFP (normoxia) performed on 5 representative HIF target genes. An average mean has been calculated from 3 independent experiments and used to generate the graph (in each experiment, each gene was analyzed in triplicate). (C) Left panel: Lactate content of culture media from HaCaT cells infected with AdGFP-18E2 or AdGFP, transfected with Ctrl or HIF-1α siRNA and grown in normoxia. The graph displays the results of one representative experiment (each reading was performed in triplicate). Data were analyzed using a one-tailed paired t test for comparison between 2 groups. Right panel: Relative HIF-1α mRNA levels in the same experiment, showing silencing by the HIF-1α siRNA.

ROS generation has an important role to play during hypoxia, when it stabilizes HIF (Hypoxia Inducible Factor) helping to promote survival and growth of cells in low oxygen environment. Under normoxia, the α subunit of HIF is hydroxylated, which serves as a recognition signal for von Hippel-Lindau ubiquitin-ligase and subsequent degradation by the proteasome [15]. Under hypoxic conditions, increased generation of ROS by the mitochondrial electron transport chain inhibits HIF-1α hydroxylation to enhance stability of the transcription factor (reviewed in 16). We thus decided to look at HIF-1α levels in HPV-18 E2-expressing cells, previously shown to accumulate ROS (Figure 4B). GFP-18E2 (m.o.i. 50) was able to stabilize HIF-1α in both normoxia (21% oxygen) and hypoxia (0.2% oxygen) when compared to GFP (Figure 6A, left panel). The western-blot of this representative experiment has been quantified using ImageQuant, and although the basal levels of HIF-1α appeared higher in hypoxia, the ratio between the HIF-1α levels of GFP-18E2 and GFP-expressing cells was similar in normoxia and hypoxia (Figure 6A, right panel). Accordingly, using Real-Time PCR, we were able to detect up-regulated transcription of 3 out of 5 representative HIF target genes (PDK1, CAIX and VEGF) in GFP-18E2-expressing cells (m.o.i. 50) in normoxia (Figure 6B). PDK1 inhibits the PDH enzyme that is responsible for conversion of pyruvate to acetyl-coA, which fuels production of NADH via the Krebs cycle to permit OXPHOS. Increase in PDK1 is thus expected to reduce OXPHOS. CAIX is involved in de-acidification processes in response to increased cellular lactate, which is the final product of glycolysis when mitochondrial respiration is impaired. Altogether, release of mitochondrial ROS, stabilization of HIF-1α, and increase in PDK1 and CAIX suggest that HPV-18 E2 impedes OXPHOS and favors certain aspects of glycolysis.

We therefore sought to test whether HPV-18 E2 increased glycolysis by measuring lactate production in normoxia. After 3 days growth in normoxia, the culture medium of GFP-18E2-expressing cells (m.o.i. 50) showed a mild but significant and reproducible increase (between 1.2 and 1.6 depending on experiments) in lactate content compared to medium of cells that expressed GFP alone (Figure 6C, left panel). GFP-expressing cells in hypoxia were used as a positive control and showed a 2-fold increase in lactate quantity compared to GFP-expressing cells in normoxic conditions (Figure S5). Figure S5 also shows that lactate increased induced by HPV-18 E2 was more obvious under hypoxic conditions because the basal levels of lactate for the control cells (expressing GFP) were higher than in normoxia. However, similarly to the HIF-1α increase, the ratios between lactate quantities in GFP-18E2 and GFP cells were about the same in normoxia and hypoxia. Importantly, we verified that the lactate increased mediated by HPV-18 E2 expression was HIF-dependent by silencing HIF1-α. In these conditions, E2 was no longer able to increase lactate production (Figure 6C, left panel), showing that HIF is required to shift metabolism towards aerobic glycolysis. The silencing of HIF-1α by the siRNA was about 90% (Figure 6C, right panel). Together, these data implicate E2 in promoting a metabolic shift from aerobic respiration to glycolysis, also known as the Warburg effect [9].

## Discussion

In this report we show that the HPV-18 E2 protein localizes to mitochondria membranes, where it modifies the cristae morphology. Although HPV-18 E2 does not induce cell death in our conditions, it augments release of mitochondrial ROS, a marker of OXPHOS dysfunction, whereas the low-risk HPV-6 E2 (which shows very low interaction to mitochondria) cannot. This ROS release correlates with HIF-1α stabilization, activation of some HIF target genes, and increase in glycolysis characterized by enhanced lactate production.

In recent years, it has become increasingly evident that many viruses target mitochondria to modify cell functions. Viruses can promote cell survival by expressing analogues of host proteins which block the intrinsic pathway of apoptosis, or can induce cell death by modifying the permeability transition pore to drive cytochrome c release [17,18,19]. In the specific case of HPV, the HPV E1^E4 protein has been shown to localize to mitochondria although no role in metabolism modulation has been demonstrated [20]. So far, only 2 viral proteins have been reported to modify cellular ROS levels by altering the OXPHOS process in mitochondria; the Hepatitis C Virus (HCV) core protein, localized at the outer mitochondrial membrane, enhances Ca^2+^ uptake and increases ROS by reducing electron flow from complex I [21]; and human Hepatitis B Virus-X protein (HBV-X) partially localizes to mitochondria and increases ROS by down-regulating the expression of enzymes involved in OXPHOS [22]. Although the exact targets of E2 at mitochondria are not identified, interaction of E2 with OXPHOS proteins from complex III, which are central mediators of mitochondrial ROS production [23], as well as with the ATP synthase, a regulator of the cristae structure [10], could provide molecular bases for both modulation of mitochondria structure and ROS release. Hepatitis viruses described above share common mechanisms with HPV as described here, since all 3 viruses are oncogenic and can impact on OXPHOS. Although HCV replication can influence ROS levels [24], and conversely, peroxide treatment of human hepatoma cells can inhibit viral replication [25], the impacts of oncogenic virus-induced ROS increase on metabolic pathways, as well as on cell proliferation and viral cycle, have yet to be investigated. In the case of HPV-18 E2, we show here that the increase of ROS correlates with HIF-1α stabilization and enhanced glycolysis.

Why would HPV, and possibly other oncogenic viruses, induce a shift from OXPHOS to glycolysis? In contrast to normal cells, cancer cells preferentially generate ATP through aerobic glycolysis followed by lactic acid fermentation in the cytosol, thus reducing the OXPHOS process [9]. This Warburg effect occurs in malignant cells even if the level of oxygen is sufficient to allow mitochondrial aerobic respiration. The selective advantage of this metabolic shift in tumor cells is still poorly understood, but a recent hypothesis postulates that enhanced glycolysis could provide a biosynthetic advantage that would better support cellular proliferation compared with OXPHOS, and that PDK1 activation, shown here to be upregulated by HPV-18 E2, may be a key activator switch (reviewed in 26). Since cells in HPV lesions exhibit higher rates of proliferation than normal cells, the E2-driven shift from OXPHOS to glycolysis could help to support increased biosynthetic requirements of infected cells. Alternatively, glycolysis may be necessary for expression of the oncogenic HPV proteins E6 and E7, since abrogation of glycolysis has been shown to drastically reduce HPV transcription [27]. Interestingly, only high-risk HPV E2 proteins seem effective in modulating mitochondria metabolism, and it is tempting to hypothesize that, by favoring glycolysis, E2 could not only support the initial acceleration of cell division (benign lesions), but may subsequently promote viral gene expression (including E6/E7 oncogenes) and carcinogenic progression. Moreover, E2 induction of pro-angiogenic VEGF could stimulate vasculogenesis of HPV tumors. These new properties of E2 are part of a cluster of novel E2 functions which incriminate high-risk E2 as key proteins in early stages of HPV-mediated carcinogenesis [5,7,8,28]. Altogether, these data raise the provocative possibility that E2 may play a role in enhancing HPV-dependent cell proliferation through modulation of host metabolism.

## Materials and Methods

### Ethics Statement

The study of clinical samples was approved by the Institutional Review Boards of the National University of Singapore (NUS-IRB 09-218).

### Cell culture, infection and transfection

C33-A and Saos-2 cells were purchased from ATCC. HaCaT cells were a gift from Norbert Fusenig [29]. Cells were grown in Dulbecco Modified Eagle Medium (DMEM) supplemented with 10% fetal calf serum (FCS). Infections with recombinant adenoviruses (Ad) were performed in plain DMEM medium for 1h with polybrene (40µg/mL) as previously described [5]. Transfection with pCG expression vectors for untagged HPV-18 E2 or ΔTAD were performed using Lipofectamine (Invitrogen). Transfections with siRNA were performed using DharmaFECT1 (Dharmacon). siRNA used: GADPH (Ctrl, D-001830-01-20, Dharmacon), HIF-1α (L-004018-00-0020, Dharmacon).

### Western-blot and immunoprecipitation (IP)

Equal amounts of proteins were separated by SDS-PAGE before transfer. Membranes were revealed using ECL Plus (Amersham Biosciences). Primary antibodies: anti-GFP (TP401; Torrey Pines Biolabs), anti-cytochrome c (ab13575; Abcam), anti-porin/VDAC (529534; Calbiochem), anti-β-tubulin (T4026; Sigma-Aldrich), anti-cdc27 (C40920; BD Biosciences), anti-HIF-1α (610959; BD Biosciences), anti-TOM20 (11802-1-AP; Proteintech).

For immunoprecipitation, GFP-Trap_A beads (ChromoTek) were used against GFP-E2 or GFP. After denaturation, eluates from the beads were loaded and a western-blot was performed using anti-UQCRC2 antibody (HPA007998; Sigma). I=input (1/50), B=beads.

### Immunofluorescence (IF) and time-lapse using MitoTracker Red

Cells were infected at m.o.i. 200. MitoTracker Red (Molecular Probes) was added to the medium 5min before washing and mounting, or imaging of living cells in time-lapse. For time-lapse, cells were infected 8 hours before beginning of the time-lapse and Hoechst 33342 (0.2 µg/ml final) was added just before starting the time-lapse to label the DNA. Pictures were taken every 40 minutes. For IF, cells were fixed with 2% paraformaldehyde, permeabilized with methanol for 30min at -20°C, washed, blocked in PBS/2% serum and incubated with homemade anti-HPV-18 E2 [4] or anti-cytochrome c (ab13575; Abcam). Nuclei were stained with DAPI. All pictures were taken with the Applied Precision DeltaVision Deconvolution microscope system and images were analyzed using SoftWoRx v4.0.0.

### Immunohistochemistry

Sections from paraffin-embedded tissues (HPV-18 CIN II and HPV-6 condyloma) were dewaxed in xylene and rehydrated through grade ethanol. Endogenous peroxidase activity was blocked by incubation in 1% hydrogen peroxide in methanol for 30 minutes. Epitope retrieval was performed by heating the sections at 121°C for 10 minutes in 10mM citrate buffer (pH 6.0). Sections were then blocked in 10% goat serum for 20 minutes and labeled with purified anti-HPV-16 E2 for 1 hour. Secondary antibodies conjugated to peroxidase-labeled dextran polymer were added for 30 minutes (Dako EnVision+ Peroxidase system). After washing in tap water, 3,3′-diaminobenzidine substrate-chromogen system (Dako) was added for color development. Sections were counterstained with hematoxylin, dehydrated, and mounted in DPX. Anti-16E2 antibody was prepared as previously described [30].

### Mass spectrometry (MS)

C33-A cells were infected with recombinant adenoviruses at m.o.i. 20 for 24 hours. After extraction (300mM NaCl, 0.5% NP-40, 50mM Tris-HCl [pH 8], 1mM EDTA, protease and phosphatase inhibitors), 20mg of total proteins were immunoprecipitated using either a two-step pull-down, with a first anti-Flag beads (A2220; Sigma-Aldrich) IP followed by elution, and a second IP with GFP-Trap_A beads from ChromoTek (1^st^ and 2^nd^ LC-MS/MS [replicates]), or a single step IP with GFP-Trap_A beads (3^rd^ LC-MS/MS). After immunoprecipitation, eluted protein complexes were separated by one-dimensional 4-12% NuPage Novex Bis-Tris Gel (Invitrogen), stained using the Colloidal Blue Staining Kit (Invitrogen) and digested with trypsin using published procedures [31]. Samples were analyzed on an EASY-nanoLC (Proxeon) coupled to an Orbitrap or Orbitrap XL (Thermo, Fisher). Survey full scan MS spectra (m/z 300–1400) were acquired with a resolution of r=60,000 at m/z 400, an AGC target of 1e6, and a maximum injection time of 500ms. The ten most intense peptide ions in each survey scan with an ion intensity >2000 counts and charge state ≥ 2 were isolated sequentially to a target value of 1e4 and fragmented in the linear ion trap by collisionally-induced dissociation using a normalized collision energy of 35%. A dynamic exclusion was applied using a maximum exclusion list of 500 with one repeat count and exclusion duration of 30sec. MS data were analyzed using X! Tandem (version TORNADO (2008.02.01.4)). X! Tandem was set up to search against an in-house database that included ncbi human database (ftp://ftp.ncbi.nih.gov/refseq/H_sapiens/H_sapiens/ARCHIVE/BUILD.37.1/protein) and ncbi HPV-6 and HPV-18 databases assuming the digestion enzyme trypsin. A fragment ion mass tolerance of 0.40 Da and a parent ion tolerance of 7.0 ppm were used. Iodoacetamide derivative of cysteine was specified as a fixed modification. Pyroglutamic acid from Glu of glutamic acid, pyroglutamic acid from Gln of glutamine, deamidation of asparagine, oxidation of methionine, and acetylation of the N terminus were specified as variable modifications. Scaffold (version Scaffold_2_05_02, Proteome Software, Inc., Portland, OR) was used to validate MS/MS-based peptide, protein identifications and spectral counting.

### Electron microscopy

HaCaT-infected cells (m.o.i. 200) on dishes were washed with PBS before fixation. Pre-fixation was performed in 2.5% glutaraldehyde solution for 1-2 hours at 4°C before washing with PBS. Post-fixation was performed with 1% Osmium tetroxide (OsO4) for 1 hour before washing with PBS or deionized water. Dehydration was obtained by a series of increasing ethanol concentrations: 50% ethanol -10min, 75% ethanol -10min, 95% ethanol -10min, 100% ethanol -10min (2 changes). Infiltration was performed with a series of araldite: acetone concentrations: 100% acetone -20min, araldite: acetone (1:1) -30min, araldite: acetone (6:1) – overnight, pure araldite -30 min, pure araldite - 1 hour in a 40°C oven, pure araldite - 1 hour in a 45°C oven, pure araldite - 1 hour in a 50°C oven. Embedding was done with fresh araldite resin. Specimens were placed into a 60°C oven and baked for 24 hours. Ultra-sectioning (100nm) was performed with a Leica Ultramicrotome and staining was obtained with uranium acetate and lead citrate. JEOL JEM1400 was used for TEM imaging.

### Flow-cytometry analyses

Etoposide (50µM) or DMSO treatments were performed 7h after infection for 21h. Half of each sample was used for cell cycle analyses while the other half was used for propidium iodide (PI) uptake assays. For cell cycle analyses, cells were fixed in 70% ethanol at -20°C overnight, and stained with PI (10μg/mL)/RNase A (10μg/mL). For PI uptake, cells were directly stained with 1µg/mL PI without fixation. Cells were analyzed on a BD LSRII flow-cytometer (BD Biosciences) using the BD FACSDiva software. Cell cycle and apoptotic sub-G1 populations were analyzed using the ModFit software.

### Isolation of mitochondria

HaCaT cells were infected at m.o.i. 200. They were fractionated using the Qproteome Mitochondria Isolation Kit (Qiagen). The crude mitochondria fraction was further purified on 30% Percoll gradient. The purified mitochondria pellet was separated into soluble and insoluble (membrane-bound proteins) fractions by alkali extraction. Briefly, after Percoll gradient, crude mitochondrial pellets were resuspended in 100µl of mitochondria storage buffer (MB, Qproteome Kit) containing 0.1M Na _2_CO_3_ (pH 11.5) and incubated on ice for 30 minutes. The suspension was centrifuged for 30 minutes at 100,000g at 4°C, and the resulting pellet (membrane fraction) was separated from the supernatant (soluble fraction) and washed in PBS twice. The pellet and supernatant were denatured in Laemmli sample buffer and analyzed by western-blot.

### Mitochondrial ROS detection by time-lapse

MitoSOX Red (Invitrogen) was used to determine ROS production by mitochondria using a Deltavision RT fluorescence microscope. The positive control cells were treated with 1mM H_2_O_2_ for 2h. Twenty-four hours post-infection, cells were stained with 5µM MitoSOX Red and Hoechst, and immediately used in time-lapse experiments for 30min at 37°C, 5% CO_2_.

### Real-time PCR

VEGF: TCTACCTCCACCATGCCAAGT, GATGATTCTGCCCTCCTCCTT
PGK1: CTGTGGGGGTATTTGAATGG, CTTCCAGGAGCTCCAAACTG
PDK1: CTGGGTAATGAGGATTTGACTGT, AAGTCTGTCAATTTTCCTCAAAGG
CAIX: ACCTGGTGACTCTCGGCTACAG, CCTCAATCACTCGCCCATTC
GLUT1: CTGGGCAAGTCCTTTGAGATG, TGGTCAGGCCGCAGTACAC
HIF-1α: CCGCTGGAGACACAATCATATC, TCCTCAAGTTGCTGGTCATCAG

### Lactate quantification

HaCaT cells were grown in normoxia (21% oxygen) for 3 days after infection (m.o.i. 50). Medium was tested for lactate concentration using the lactate fluorometric assay kit (Ab65330; Abcam). For silencing of HIF-1α cells were transfected with siRNAs immediately after infection.

## Supporting Information

Table S1 **Complete list of mitochondrial proteins interacting with HPV-18 E2.** C33-A cells were infected with recombinant adenoviruses expressing Flag-GFP-18E2 or Flag-GFP at m.o.i. 20 (for the 2 first experiments). After TAP immunoprecipitation (double IP), bound proteins were analyzed by mass spectrometry (LC-MS/MS). The third LC-MS/MS compared GFP-ΔTAD with GFP-18E2 and GFP-6E2 from single IP (m.o.i. 20). The table shows spectral counting for mitochondrial proteins found to interact with HPV-18 E2 in the 3 LC-MS/MS experiments. Specific HPV-18 E2 partners appear in red, whereas proteins interacting with both HPV-18 and HPV-6 E2 appear in blue.
(PDF)

Figure S1 **E2 does not co-localize with Endoplasmic Reticulum (ER) or Golgi.** AdGFP-18E2-infected cells labeled with anti-calreticulin (ER) and anti-GM130 (Golgi) antibodies, and revealed by secondary antibodies coupled to Alexa 568.
(PDF)

Figure S2 **Real-Time PCR showing the relative quantities of E2 transcripts in cells expressing the full HPV-18 genome compared to cells infected with increasing m.o.i. of the GFP-E2 adenovirus.** Transcripts were analyzed 24h after infection and 24h or 48h after transfection of the HPV-18 genome as indicated. Non-infected cells (E2 0) and transfection with GFP were used as negative controls.
(PDF)

Figure S3 **Flow-cytometry quantification of mitochondrial ROS release in cells expressing HPV-18 GFP-E2.** Thirty hours post-infection, cells were treated with 5µM MitoSOX Red for 10 min at 37°C, washed and processed for IF or flow-cytometry. For flow-cytometry, unfixed cells were counterstained with Dapi (1µg/mL) and gated on the Dapi-negative population to select only living cells for further analyzes. H_2_O_2_ was used as a positive control for ROS production.
(PDF)

Figure S4 **Cytochrome c is not released from mitochondria after expression of GFP-18E2 at m.o.i. 200.** Western-blot analyses after fractionation of extracts from HaCaT cells infected with AdGFP-ΔTAD and AdGFP-18E2 (m.o.i. 200). Nuc: nuclear fraction, Cyt: cytoplasmic fraction, Mito: crude mitochondrial fraction after Percoll gradient.
(PDF)

Figure S5 **Lactate content of culture media from HaCaT cells infected with AdGFP-18E2 or AdGFP and grown in normoxia (21% O_2_) or hypoxia (0.2% O_2_) for 3 days.** The Y axis represents the ratio for each value relative to the GFP value obtained at 21% O_2_. The graph displays the mean of 3 independent experiments where each reading was performed in triplicate. Data were analyzed using a one-tailed paired t test for comparison between 2 groups.
(PDF)
