# Supplementary material for: Localization of HPV-18 E2 at Mitochondrial Membranes Induces ROS Release and Modulates Host Cell Metabolism
Source: PLoS One. 2013 Sep 24;8(9):e75625. doi: 10.1371/journal.pone.0075625 (PMC3782431; doi:10.1371/journal.pone.0075625)
Supplement: Table S1 — Complete list of mitochondrial proteins interacting with HPV-18 E2. C33-A cells were infected with recombinant adenoviruses expressing Flag-GFP-18E2 or Flag-GFP at m.o.i. 20 (for the 2 first experiments). After TAP immunoprecipitation (double IP), bound proteins were analyzed by mass spectrometry (LC-MS/MS). The third LC-MS/MS compared GFP-ΔTAD with GFP-18E2 and GFP-6E2 from single IP (m.o.i. 20). The table shows spectral counting for mitochondrial proteins found to interact with HPV-18 E2 in the 3 LC-MS/MS experiments. Specific HPV-18 E2 partners appear in red, whereas proteins interacting with both HPV-18 and HPV-6 E2 appear in blue. (PDF) [file pone.0075625.s001.pdf]

Gene name and pathway		gi number	1st LC-MS/MS		2nd LC-MS/MS		3rd LC-MS/MS		
			GFP	GFP-18E2	GFP	GFP-18E2	GFP-ΔTAD	GFP-18E2	GFP-6E2
**Respiratory chain**									
UQCRC2	ubiquinol-cytochrome c reductase core protein II	gi|50592988	0	46	0	15	15	34	9
ATP5O	mitochondrial ATP synthase, O subunit precursor	gi|4502303	0	26	0	15	3	12	8
UQCRFS1	ubiquinol-cytochrome c reductase, Rieske iron-sulfur polypeptide 1	gi|163644321	0	10	0	2	0	6	3
CYC1	cytochrome c-1	gi|21359867	0	18	0	3	0	2	1
COX2	cytochrome c oxidase subunit II	gi|17981856	0	8	0	2	0	4	3
**Krebs cycle**									
IDH2	isocitrate dehydrogenase 2 (NADP+), mitochondrial precursor	gi|28178832	0	4	0	4	2	11	3
**Protein synthesis/DNA**									
ATAD3A	ATPase family, AAA domain containing 3A	gi|42476028	0	12	0	36	11	34	5
POLRMT	mitochondrial DNA-directed RNA polymerase precursor	gi|110618253	0	6	0	19	1	5	2
MRPL43	mitochondrial ribosomal protein L43 isoform b	gi|28872732	0	6	0	2	0	3	0
**Mitochondrial import**									
DNAJA1	DnaJ (Hsp40) homolog, subfamily A, member 1	gi|4504511	0	66	0	42	14	37	8
SLC25A11	solute carrier family 25 (mitochondrial carrier; oxoglutarate carrier), member 11	gi|21361114	0	16	0	16	2	6	3
SLC25A10	solute carrier family 25 (mitochondrial carrier; dicarboxylate transporter), member 10	gi|20149598	0	18	0	17	0	5	0

Table S1
