# Supplementary figures and images for: Localization of HPV-18 E2 at Mitochondrial Membranes Induces ROS Release and Modulates Host Cell Metabolism
Source: PLoS One. 2013 Sep 24;8(9):e75625. doi: 10.1371/journal.pone.0075625 (PMC3782431; doi:10.1371/journal.pone.0075625)

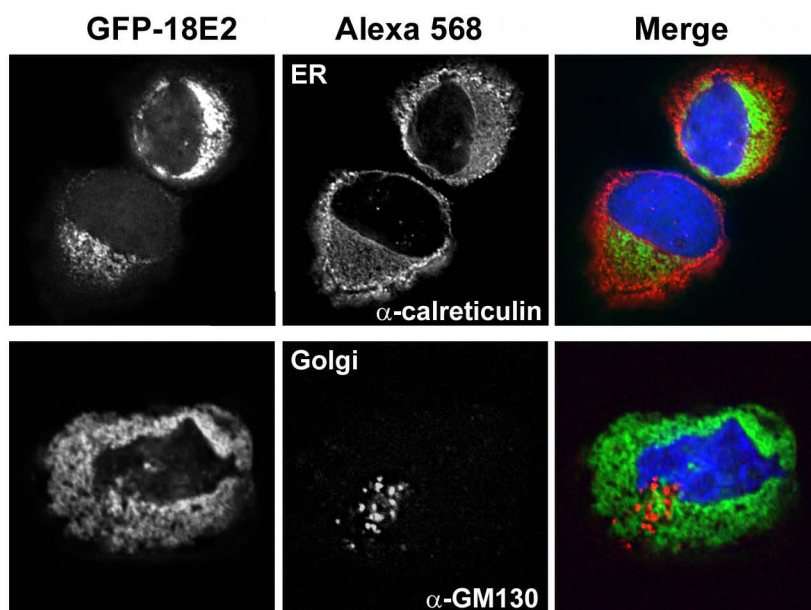

**Figure S1**

Supplement: Figure S1 — E2 does not co-localize with Endoplasmic Reticulum (ER) or Golgi. AdGFP-18E2-infected cells labeled with anti-calreticulin (ER) and anti-GM130 (Golgi) antibodies, and revealed by secondary antibodies coupled to Alexa 568. (PDF) [file pone.0075625.s002.pdf]

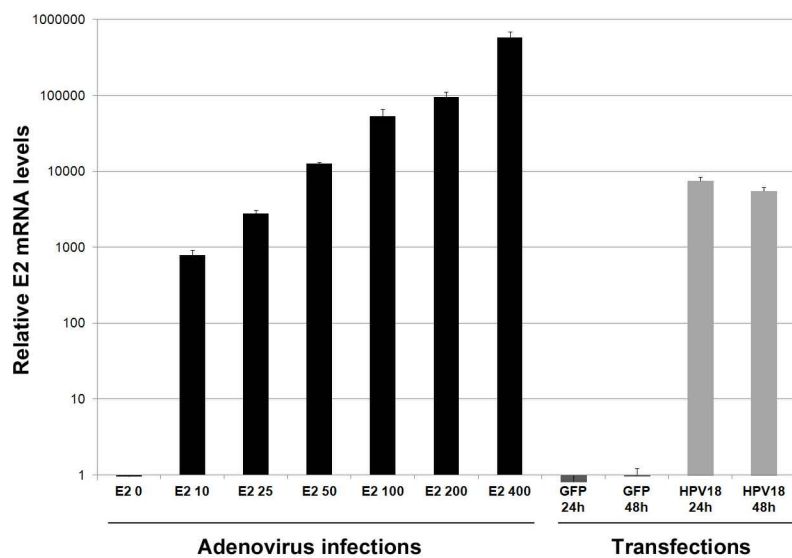

Figure S2

Supplement: Figure S2 — Real-Time PCR showing the relative quantities of E2 transcripts in cells expressing the full HPV-18 genome compared to cells infected with increasing m.o.i. of the GFP-E2 adenovirus. Transcripts were analyzed 24h after infection and 24h or 48h after transfection of the HPV-18 genome as indicated. Non-infected cells (E2 0) and transfection with GFP were used as negative controls. (PDF) [file pone.0075625.s003.pdf]

**A**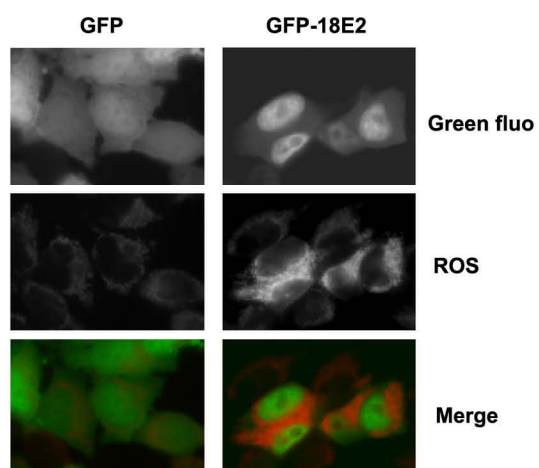**B**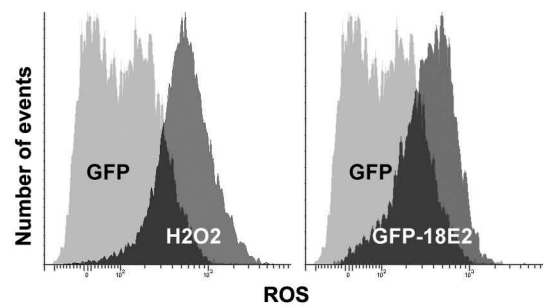**Figure S3**

Supplement: Figure S3 — Flow-cytometry quantification of mitochondrial ROS release in cells expressing HPV-18 GFP-E2. Thirty hours post-infection, cells were treated with 5µM MitoSOX Red for 10 min at 37°C, washed and processed for IF or flow-cytometry. For flow-cytometry, unfixed cells were counterstained with Dapi (1µg/mL) and gated on the Dapi-negative population to select only living cells for further analyzes. H2O2 was used as a positive control for ROS production. (PDF) [file pone.0075625.s004.pdf]

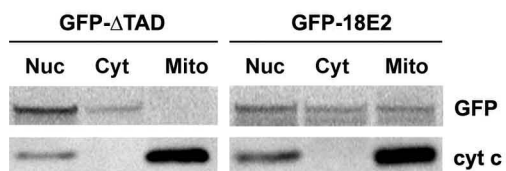

**Figure S4**

Supplement: Figure S4 — Cytochrome c is not released from mitochondria after expression of GFP-18E2 at m.o.i. 200. Western-blot analyses after fractionation of extracts from HaCaT cells infected with AdGFP-ΔTAD and AdGFP-18E2 (m.o.i. 200). Nuc: nuclear fraction, Cyt: cytoplasmic fraction, Mito: crude mitochondrial fraction after Percoll gradient. (PDF) [file pone.0075625.s005.pdf]

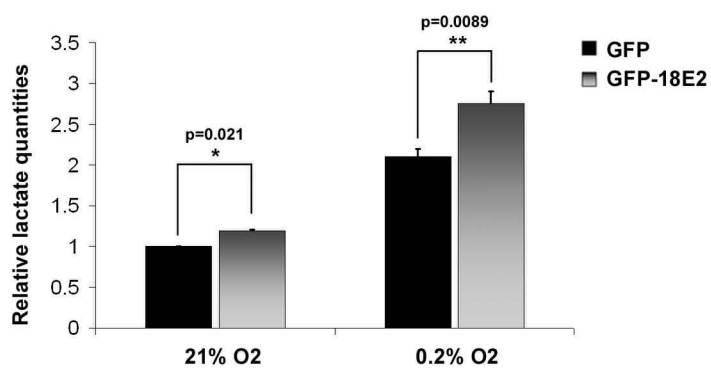

**Figure S5**

Supplement: Figure S5 — Lactate content of culture media from HaCaT cells infected with AdGFP-18E2 or AdGFP and grown in normoxia (21% O2) or hypoxia (0.2% O2) for 3 days. The Y axis represents the ratio for each value relative to the GFP value obtained at 21% O2. The graph displays the mean of 3 independent experiments where each reading was performed in triplicate. Data were analyzed using a one-tailed paired t test for comparison between 2 groups. (PDF) [file pone.0075625.s006.pdf]
